# *BnALMT7-A4* encodes an aluminium-activated malate transporter that enhances aluminium tolerance in both *Brassica napus* L. and *Arabidopsis thaliana*

**DOI:** 10.3389/fpls.2025.1710318

**Published:** 2026-01-28

**Authors:** Xulyu Cao, Aihui Liu, Xiaoyong Zhang, Kaiyang Liu, Lanyang Ren, Can Liu, Juliet C. Coates, Nannan Li

**Affiliations:** 1 College of Resources and Environment, Academy of Agricultural Sciences, Key Laboratory of Efficient Utilization of Soil and Fertilizer Resources, Southwest University, Chongqing, China; 2 School of Biosciences, University of Birmingham, Birmingham, United Kingdom; 3 Yibin Academy of Southwest University, Yibin, Sichuan, China

**Keywords:** *Brassica napus*, aluminium tolerance, *ALMT*, root, hairy root

## Abstract

**Introduction:**

Aluminium (Al) toxicity in acidic soil is a key limiting factor of agricultural productivity and sustainability. ALUMINIUM-ACTIVATED MALATE TRANSPORT (*ALMT*) homologs regulate responses to acidic soil conditions by releasing malate to chelate toxic Al^3+^ ions, thus also reducing the ability of Al^3+^ to bind to inorganic phosphate (Pi) and lower Pi bioavailability. In rapeseed (*Brassica napus*), *BnALMT1* and *BnALMT2* mitigate Al stress. However, function of *BnALMT7*, in the same clade as *BnALMT1*, remains unknown. Here we identified and characterised ALMT7 homologs (*BnALMT7-A4* and *BnALMT7-C4*) in rapeseed, and used one homolog, *BnALMT7-A4*, to engineer both Al-tolerant Arabidopsis plants and Al-tolerant Brassica hairy roots, and to understand the mechanism by which this Al-tolerance is conferred by *BnALMT7-A4*.

**Methods:**

*BnALMT7-A4* and *BnALMT7-C4* gene expression was characterised using qRT-PCR and promoter activity was assayed with a *pBnALMT7-A4::GUS* fusion. The protein structres were assessed by modelling and BnALMT7-A4 was characterised using a *BnALMT7-A4-GFP* fusion protein and a split luciferase assay. Transgenic Arabidopsis and rapeseed hairy root lines overexpressing *BnALMT7-A4* were generated to investigate the function of *BnALMT7-A4* under Al stress, including via transcriptomic analysis.

**Results:**

*BnALMT7-A4* and *BnALMT7-C4* were predicted to be transmembrane proteins. *BnALMT7-A4* showed the greatest similarity to *Arabidopsis AtALMT7*, localised to the plasma membrane and formed homodimers. In addition to their constitutive expression in flowers and siliques, both *BnALMT7-A4* and *BnALMT7-C4* were significantly induced by Al treatment in roots. The promoter of *BnALMT7-A4* was specifically active in the root vascular system. Phenotypic analysis of overexpression lines generated in both Arabidopsis plants and Brassica hairy roots revealed that *BnALMT7-A4* promoted root growth, with less Al accumulation occurring in the root tips of transgenic plants. Transcriptomic analysis showed that overexpression of *BnALMT7-A4* resulted in upregulation of genes response to oxidative stress and downregulation of genes involved in detoxification in the presence of Al.

**Discussion:**

We have identified a putative aluminium-activated malate transporter, *BnALMT7-A4*, that is induced by Al treatment in roots. We engineered Arabidopsis and Brassica overexpressing *BnALMT7-A4* to generate Al-tolerant plants with improved root growth and reduced Al accumulation in root tips. Transcriptomic analysis of the Al-tolerant Brassica roots demonstrated modification of stress- and toxicity-specific gene expression. Thus, we have discovered a new way of making rapeseed, an important crop, more tolerant to Al stress.

## Introduction

Acidic soils, characterized by a pH lower than 5.5, encompass approximately 50% of the world’s potential arable land, which significantly constrains crop growth and economic yield (Chauhan et al., 2021; Ranjan et al., 2021). Among the challenges caused by acidic soils, widespread heavy-metal toxicities and mineral nutrient deficiencies, particularly Aluminium (Al) toxicity and phosphorus deficiency, emerge as the most severe (Delhaize and Ryan, 1995; Chauhan et al., 2021; Yu et al., 2023). Aluminium comprises 7% of the Earth’s crust and is the most abundant metal and third most abundant element (Exley, 2009). Al is generally non-toxic to plants when present in the form of aluminosilicate or aluminium oxide (Rahman et al., 2018; Botté et al., 2022). However, at soil pH values below 5, the dissolution of silicate releases Al^3+^, inhibiting root system growth and reducing crop yield (Delhaize and Ryan, 1995; Rahman et al., 2018). Moreover, released Al^3+^ complexes with phosphorous (P), reducing available P for plant nutrition (Dabravolski and Isayenkov, 2023). Soil acidification is a particular problem in China and impacts cultivation of key crops (Guo et al., 2010). *Brassica napus* (oilseed rape, rapeseed or canola) is a key crop for oil and animal feed that presents a particular concern due to cultivation in the Yangtze river basin where acidification and Al toxicity have become limiting factors for crop productivity (Gao et al., 2021; Li et al., 2023).

Plants have evolved various strategies to cope with Al^3+^ toxicity, including the secretion of organic acids (OAs, including malate, citrate and oxalate) and phenolics to chelate Al^3+^. Plants also transport toxic Al compounds into vacuoles for sequestration, fix Al^3+^ in the cell wall to minimize cellular entry, induce the secretion of proteins such as metallothionein and phytochelatin that bind Al, enhance antioxidant enzyme activity, and modify the rhizosphere pH (Ofoe et al., 2023; Ur Rahman et al., 2024). OAs secreted from roots is the first line of defense upon Al exposure and the ALUMINIUM-ACTIVATED MALATE TRANSPORT (*ALMT*) family has been identified and studied in several different species.

The first Al-resistance gene to be identified was *TaALMT1*, from wheat (Sasaki et al., 2004). *Ta*ALMT1 mediates Al^3+^-dependent malate efflux from cells and improves Al-resistance when expressed in barley, tobacco and the model plant *Arabidopsis* (Delhaize et al., 2004; Sasaki et al., 2004; Pereira et al., 2010; Ryan et al., 2011). Expression of the *Arabidopsis* homologue *AtALMT1* is induced by Al^3+^ and *At*ALMT1 increases cellular malate efflux (Hoekenga et al., 2006). *AtALMT1* is expressed in the root epidermis and an *Arabidopsis almt1* mutant is hypersensitive to Al treatment (Hoekenga et al., 2006). The closest crop plants to *Arabidopsis* are *Brassica* species. In *Brassica napus*, *BnALMT1* and the very closely related *BnALMT2* are induced in roots (but not shoots) by Al stress and they increased malate efflux and Al-tolerance in heterologous systems (Ligaba et al., 2006). Similarly, in cabbage (*Brassica oleracea*), *BoALMT1* is root-enriched, induced by Al and promotes malate secretion and Al tolerance in transgenic *Arabidopsis* (Zhang et al., 2018). In *Arabidopsis*, Al-induced expression of *AtALMT1* requires the zinc-finger transcription factor SENSITIVE TO PROTON RHIZOTOXICITY1 (STOP1) (Iuchi et al., 2007). *AtALMT1* expression is also regulated by other signals and stresses including low pH and salt (Dabravolski and Isayenkov, 2023). Further crop ALMT1 homologs have similar functions in response to Al stress, including those from false flax (*Camelina sativa*), soybean (*Glycine max*), white lupin (*Lupinus albus*), alfalfa (*Medicago sativa*) and rye (*Secale cereale*) (Collins et al., 2008; Liang et al., 2013; Park et al., 2017; Zhou et al., 2020; Jin et al., 2024).

Malate has plant functions independently from responding to Al stress. For example, apple (*Malus domestica*) *Md*ALMT9, tomato (*Solanum lycopersicum*) *Sl*ALMT9 and grape (*Vitis vinifera*) *Vv*ALMT9 all formed malate channels on the vacuolar membrane, mediating malate transport from the cytosol to the vacuole to regulate fruit acidity (Ma et al., 2015; Li et al., 2020a, 2024; Ye et al., 2021). *Vv*ALMT2 is a root-expressed *ALMT* that also transports nitrate and other anions with expression of *VvALMT2* in *Arabidopsis* mitigating the effects of salt stress (Wu et al., 2024). *SlALMT5* was found to affect malate content and OA composition in tomato seeds (Sasaki et al., 2016). In rice (*Oryza sativa*), *OsALMT7*, a homolog of AtALMT13 was expressed in vascular tissues and played a role in maintaining panicle size and grain yield by regulating malate transport in the panicles, which was independent of Al activation (Heng et al., 2018).

In total, *Arabidopsis* has 14 *ALMT* genes (Dreyer et al., 2012; Sharma et al., 2016; Dabravolski and Isayenkov, 2023; Jaślan et al., 2023). *AtALMT2*, *AtALMT7* and *AtALMT8* fall into the same clade as *AtALMT1* but have not yet been characterized. In more distant clades from *AtALMT1*, *AtALMT3* is upregulated by low phosphate in root hair cells (Maruyama et al., 2019), while *AtALMT4*, *AtALMT6*, *AtALMT9* and *AtALMT12–14* have, with non-redundant functions in the regulation of stomatal closure and stomatal opening (Meyer et al., 2010, 2011; De Angeli et al., 2013; Eisenach et al., 2017; Medeiros et al., 2018; Jaślan et al., 2023).

With a more complex (tetraploid) genome than *Arabidopsis*, *Brassica napus* possesses 39 *ALMT* genes that have been analyzed in the context of phosphate starvation (Din et al., 2021). Several large-scale screening studies have identified over 200 potential loci for *Brassica napus* aluminium tolerance (Gao et al., 2021; Li et al., 2023; Zhou et al., 2024). However, none of these genes have been confirmed experimentally and none are in the *ALMT* family. Plant ALMTs have been subject to structural analysis, identifying ~6 transmembrane domains and residues critical for the sensing of Al, malate and other signals (reviewed in Dabravolski and Isayenkov, 2023). *At*ALMT1 forms homodimers in the plasma membrane, similarly to *At*ALMT9, *Os*ALMT7 and *Gm*ALMT12 (Qin et al., 2022; Wang et al., 2022; Zhou et al., 2022; Qian et al., 2024). Amongst the 14 *Arabidopsis* ALMTs, *At*ALMT7, in the *At*ALMT1 clade, is unique in possessing an insertion of extra amino acid residues C-terminal to the 6^th^ transmembrane domain and before the key Al sensing residues (Dabravolski and Isayenkov, 2023). To the best of our knowledge, the biological function of this insertion have never been tested experimentally.

In this study, we sought to characterize *ALMT7* homologue(s) in *Brassica napus*. Furthermore, we engineered *Arabidopsis* plants and *Brassica* roots with overexpression of one *BnALMT7* homologue, *BnALMT7-A4*, which possesses the same insertion as *AtALMT7*. This led to increased resistance to Al-toxicity in both systems. Using transcriptome analysis in Arabidopsis, we defined possible mechanisms by which *Bn*ALMT7 functioned to mitigate Al stress. Thus, our study provides new insights into the mechanisms of Al tolerance in the Brassicaceae and provides possible new routes to generating Al-tolerance in a key crop.

## Materials and methods

### Plant materials and growth conditions

*Brassica napus* L. cultivar “ZS11” was obtained from the Oil Crop Research Institute of the Chinese Academy of Agricultural Science (Wuhan, China) and used for gene amplification and hairy root transformation. All *Arabidopsis* mutant/transgenic lines are in the Columbia (Col-0) ecotype. The T-DNA insertion mutant *almt1* (At1g08430, SALK_009629) was kindly provided by Prof Chaofeng Huang, CEMPS (Center for Excellence in Molecular Plant and Science, Shanghai). Wild type *Nicotiana benthamiana* was used for luciferase assays. All plants were grown in the growth chamber of the Academy of Agricultural Science in Southwest University (Chongqing, China) under a 14h light (25°C)/10h dark (23°C) photoperiod.

### Bioinformatic analysis of *BnALMT7*s

Amino acid sequences of *BnALMT7*s homologs from different species were obtained from Phytozome 13 (https://phytozome-next.jgi.doe.gov) (Goodstein et al., 2012). Multiple sequence alignments were conducted using SEAVIEW with clustalo and a phylogenetic tree was constructed with the neighbor-joining method (Gouy et al., 2010). The multiple sequence alignment was visualized with GeneDoc (Nicholas et al., 1997). The deep TMHMM website (https://services.healthtech.dtu.dk/services/DeepTMHMM-1.0/) was used to predict transmembrane domains (Hallgren et al., 2022). The Alphafold3 server (https://deepmind.google/technologies/alphafold/alphafold-server/) was used to predict protein structure and protein interaction (Jumper et al., 2021; Abramson et al., 2024; Varadi et al., 2024).

### Plasmid construction and plant transformation

To generate overexpression lines and complementation lines of *BnALMT7-A4* (BnaA04g15700D), the coding sequence of *BnALMT7-A4* was constructed into a plant binary C-terminal GFP fusion vector (Pcx-DG), driven by the *CaMV 35S* promoter (Chen et al., 2009). The plasmid was then transformed into *Agrobacterium tumefaciens* GV3101, which was used for floral dip transformation (Clough and Bent, 1998) to generate overexpression and complementation lines in Col-0 and the *Atalmt1* mutant, respectively. For overexpressing *BnALMT7-A4* in hairy roots, *BnALMT7-A4*, driven by a *CaMV 35S* promoter, was introduced into the pNmGFPer vector, in which GFP was driven by another *CaMV 35S* promoter and used as an indicator of positive transformation. To generate the *pBnALMT7-A4*::*GUS* construct, the 2kb upstream of the *BnALMT7-A4* gene was amplified and cloned into the pCAMBIA1305.1 vector containing the *uidA* gene encoding β-glucuronidase (GUS) (Das et al., 2020). The plasmid was transformed into *Agrobacterium rhizogenes* MSU440 for hairy root transformations. Primer sequences used in plasmid construction are listed in **Supplementary Table S1**.

### Subcellular localization in *Nicotiana benthamiana*

The GFP::*BnALMT7-4A* fusion construct was transformed into *Agrobacterium tumefaciens* GV3101 with RNA silencing repressor P19, which was then co-infiltrated in tobacco leaves with plasma membrane maker pCr40 (mCherry). After infiltration, plants were incubated in the dark at 25°C for 12 h, then cultured in a 14h light (25°C)/10h dark (23°C) photoperiod for 36 h. The green and red fluorescence was observed using confocal microscope (Zeiss, LSM780, Germany). The empty vector (35S-GFP) was used as negative control.

### Hairy root transformation

Seeds of *Brassica napus* L. cultivar “ZS11” were sterilized and germinated on the half-strength Murashige and Skoog (MS) medium (PM1011-50L, Coolaber) (pH 5.8) in magenta pots containing 1% sucrose and 1% agar (8211GR500, Neofroxx). The magenta pots were incubated in dark at 24°C for 3~4 d until the etiolated cotyledons grew out. Then the pots were grown in light at 24°C for 2 d, and green cotyledons were excised and immersed in induction media (half-strength MS liquid, 1% sucrose, 300 µM acetosyringone) with *Agrobacterium rhizogenes* MSU440 (OD_600_, 0.1) for 15 min. The cotyledons were then transferred onto sterilized filter papers placed in cocultivation agar plates (half-strength MS liquid, 1% sucrose, 1% agar, 100 µM acetosyringone) and incubated in the dark at 24°C for 36 h. The cotyledons were transferred onto hairy root agar plates (half-strength MS liquid, 1% sucrose, 300 mg/L timentin, 0.4% agar). The plates were incubated at 24°C under 16 h light/8h dark, and hairy roots grew in 15 d. For screening of positive transformations, hairy roots with green fluorescence under a hand-held lamp (LUYOR-3415RG, USA) were selected, which were used for further Al staining and Al tolerance evaluation.

### Al tolerance evaluation

*Arabidopsis* seeds of Col-0, *almt1*, heterologous overexpression and complementation lines of *BnALMT7-A4* were sterilized and germinated on the half-strength MS plates (pH 5.8) containing 1% sucrose and 1% agar. The plates were incubated at 4°C for 2 d to vernalize the seed before growing seedlings vertically in a growth chamber with a 16 h/8 h day/night cycle at ~20°C to 22°C for 3 d. Seedlings with similar root lengths (~0.5 cm) were transferred to an agar plate containing 1 mM CaCl_2_, 1% sucrose and 1% agar with 0 or 50 µM AlCl_3_ for 5 d. The plates were scanned with an Espon Perfection V800 Flatbed Photo Scanner and root lengths were measured with ImageJ (Schneider et al., 2012).

To evaluate Al tolerance of *BnALMT7-A4* overexpression hairy roots, positive transformed hairy roots with similar length and width were excised and transferred onto an agar plate containing 1 mM CaCl_2_, 1% sucrose and 1% agar with 0 or 50 µM AlCl_3_ for 3 d. The plates then were scanned and analyzed as described above.

### RNA extraction and quantification reverse transcription PCR analysis

To analyze the expression pattern of the *BnALMT7* genes, roots, stems, leaves, flowers and siliques of *Brassica napus* L. cultivar “ZS11” were collected for RNA extraction. To compare time-series expression of *BnALMT7*s, 2-week-old rapeseed plants were pretreated with a 0.5mM CaCl_2_ solution at pH 4.8 for 12 h and then treated with the same CaCl_2_ solution (with or without 50 µM AlCl_3_). The roots were sampled at 0h, 12h, 24h, 36h and 48h for RNA isolation. Total RNA was extracted using EZ-10 DNAaway RNA Mini Preps Kit (Sangon Biotech, B618133-0050). The complementary DNA (cDNA) was synthesized with PrimeScript™ RT Reagent Kit with gDNA Eraser (Takara, Japan), which was used for both amplification of full-length *BnALMT7-A4* and qPCR analysis of *BnALMT7*s.

The qRT-PCR was conducted using GoTaq qPCR Master Mix (Promega, USA) on an ABI Prism 7900 Sequence Detection System as described previously (Li et al., 2020b). The housekeeping gene *ACTIN* (BnaA09g04490D) was used as the internal reference. At least three biological replicates were performed for each gene. The primer sequences used in qRT-PCR are listed in **Supplementary Table S2**.

### GUS histochemical staining

Hairy root GUS staining was conducted as previously described (Peng et al., 2024). Briefly, hairy roots were immersed in a 1 mM CaCl_2_ solution for 12 h before transferring to a new 1 mM CaCl_2_ solution with either 0 or 50 μM AlCl_3_ for 6 h. The samples were then soaked in GUS staining buffer (50 mM Sodium phosphate buffer, pH 7.0, 0.1% Triton X-100, 2 mM K_3_Fe(CN)_6_, 2 mM K_4_Fe(CN)_6_, EDTA, pH 8.0, 2 mM 5-bromo-4-chloro-3-indolyl β-D-glucuronide) and placed under vacuum for 15 min before incubating at 37 °C for 3 h. Stained samples were mounted on slides with 50% glycerol and imaged with an Espon Perfection V800 Flatbed Photo Scanner.

For histological sections, samples were stained as described above and embedded in 3% agarose. Cross sections (75 μm) were obtained with a vibratome (Leica VT1000S), which were observed and documented under a Leica DM2000 LED microscope.

### Al staining

Al accumulation in the root tips of *Arabidopsis* roots and *B. napus* hairy roots was determined by using Eriochrome Cyanine R (ECR) staining to detect metal cations. Roots were immersed in a solution containing 0.5mM CaCl_2_ at pH 4.8 for 6 h and then transferred into the same solution supplemented with 0 or 50 µM AlCl_3_ for 12 h. The roots were washed with distilled water before staining with 0.1% Eriochorome Cyanine R (E10037-5G, Psaitong) for 10 min. The roots were then washed with distilled water for 10 min three times and mounted on slides in 50% glycerol, then scanned with an Epson Perfection V800 Flatbed Photo Scanner.

### Split firefly luciferase assay

Split firefly luciferase assays were performed by *Agrobacterium* infiltration in *N. benthamiana* leaves as previously described (Wang et al., 2021). In brief, the full-length coding sequences of *BnALMT7-A4* with or without a stop codon were introduced into *pCAMBIA1300-cLUC* and *pCAMBIA1300-nLUC*, forming *BnALMT7-A4-cLUC* and *BnALMT7-A4-nLUC* constructs, respectively (Yan et al., 2020). The constructs were transformed into *Agrobacterium tumefaciens* GV3101 with RNA silencing repressor P19, which were then co-infiltrated into *N. benthamiana* leaves. After infiltration, plants were incubated in the dark at 25°C for 12 h, then cultured in a 14h light (25°C)/10h dark (23°C) photoperiod for 36 h. For chemiluminescence signal imaging, *N. benthamiana* leaves were infiltrated with 0.5 mM luciferin (ST196-100mg, Beyotime) to activate chemiluminescence signals, which were captured by a Chemiluminescent Image System (Tanon-5200). The primer sequences used in the luciferase complementation assay are listed in **Supplementary Table S2**.

### Hairy root RNA-seq and data analysis

For RNA-seq analysis, *BnALMT7-A4* overexpression hairy roots and corresponding negative transformants were prepared, and cultured on agar plates (pH 5.0) containing 0.5mM CaCl_2_, 1% agar, 0 or 50 µM AlCl_3_ for 3 d before harvesting. Total RNA was extracted from hairy root tissues by using TRIzol reagent according to the manufacturer’s instructions (Qiagen, Germany). The RNA sequencing libraries were constructed following Illumina Standard mRNA Prep, Ligation (San Diego, CA), and sequenced with NovaSeq X Plus platform (PE150) (Majorbio, Shanghai, China). Raw data were trimmed and filtered with fastp (https://github.com/OpenGene/fastp) to acquire clean reads, which were mapped to the reference genome (https://yanglab.hzau.edu.cn/BnIR/germplasm_info?id=ZS11.v0) with orientation mode in HISAT2 (http://ccb.jhu.edu/software/hisat2/index.shtml). The mapped reads of each sample were assembled with StringTie software. For expression level analysis, RSEM (http://deweylab.github.io/RSEM) was used for gene abundance quantification, after calculation of each transcript with the transcripts per million reads (TPM) method. Differential expression analysis was performed using DESeq (http://bioconductor.org/packages/stats/bioc/DESeq2) with FDR<0.05. The GO functional enrichment analysis and KEGG pathway analysis were performed by Goatools (https://github.com/tanghaibao/GOatools) and python scripy package (https://scipy.org/install/) with a corrected p value < 0.05, respectively. The data were analyzed and plotted on the online platform of Majorbio Cloud Platform (www.Majorbio.com) and Chiplot (https://www.chiplot.online/) (Ren et al., 2022; Han et al., 2024).

## Results

### Identification and sequence analysis of *BnALMT7*s

**Figure 1:**
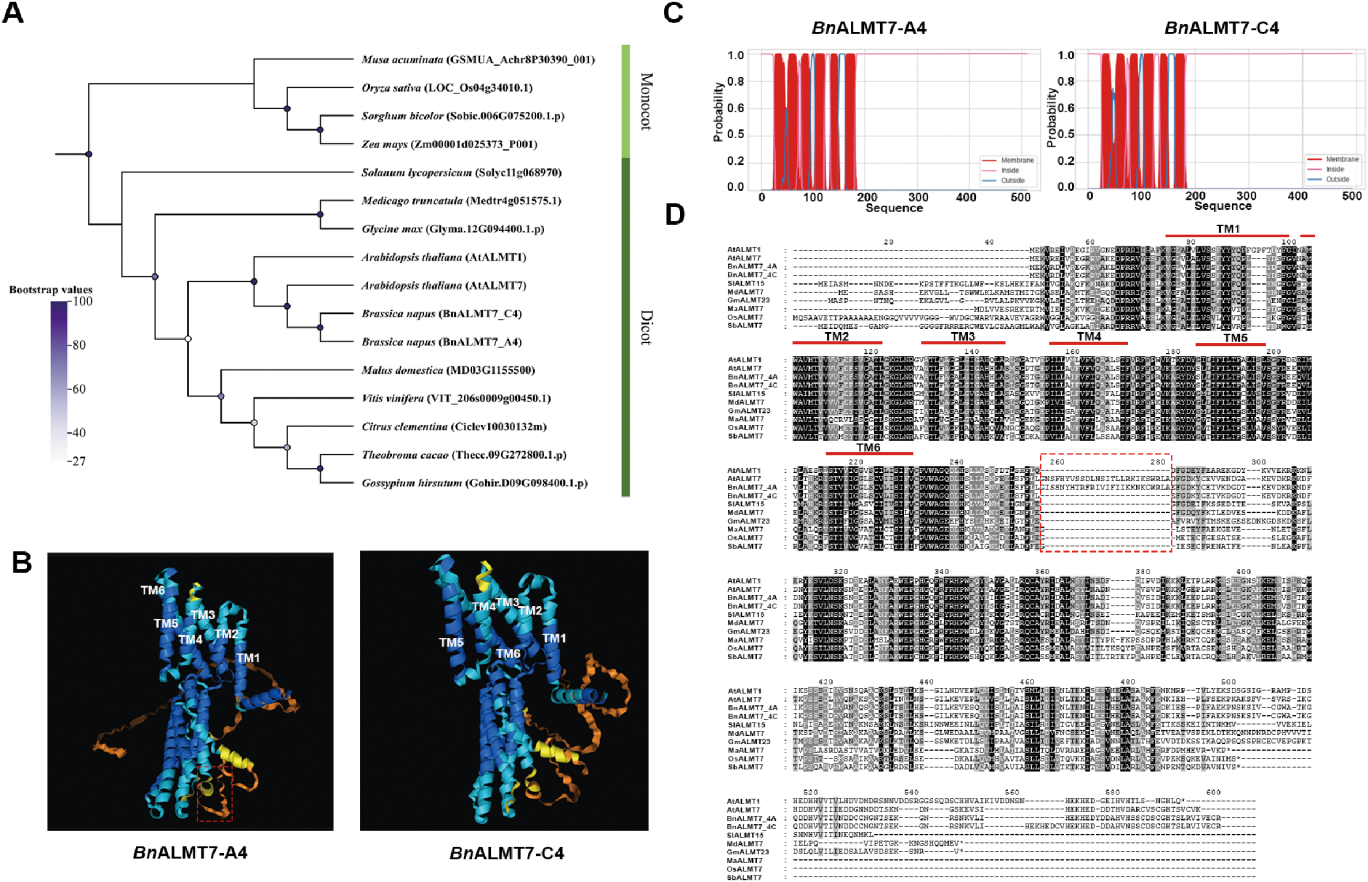
Characterisation of BnALMT7s. **(A)** Phylogenetic analysis of amino acid sequences among known or unknown ALMT7s from different species. **(B)** Structural prediction of BnALMT7-A4 and BnALMT7-C4 proteins with Alphafold server 3.0. Different colors represent different scores of Predicted Local Distance Difference Test (pLDDT). Blue indicates the residue has a very high confidence (pLDDT >90), cyan indicates a high confidence (90>pLDDT>70), orange indicates a low confidence (>70pLDDT>50) and yellow represents a very low confidence (pLDDT<50). The red box highlighted the unique motif of BnALMT7-A4. **(C)** Prediction of transmembrane domains in BnALMT7s. Red indicates transmembrane domain, pink indicates residues are inside of cell and blue indicates residues are outside of cell. **(D)** Multiple sequences alignment of BnALMT7s with other ALMT7 homologs. The red bars indicate transmembrane domains, and red box highlighted the unique motif.

A previous study identified 39 *BnALMT*s in *Brassica napus* named according to the sequences of 14 *AtALMT*s from *Arabidopsis* (Din et al., 2021). Phylogenetic analysis revealed that there were two *BnALMT7* homologues in *Brassica napus*, named *BnALMT7-A4* (*BnALMT7.2*, BnaA04g15700D) and *BnALMT7-C4* (*BnALMT7.1*, BnaC04g38980D) (Din et al., 2021; **Supplementary Figure 1**). BLAST analysis revealed that the amino acid sequences of *Bn*ALMT7-A4 and *Bn*ALMT7-C4 share 78% and 81.2% similarity to *At*ALMT7 and 61% and 64.3% to *At*ALMT1, respectively. The highly similar (92.6%) sequences of *Bn*ALMT7-A4 and *Bn*ALMT7-C4 matched with the same genes when BLAST was carried out against the genomes of other species, including *Solanum lycopersicum* (*SlALMT15*, Solyc11g068970), *Medicago truncatula* (*MtALMT7*, Medtr4g051575.1), *Glycine max* (*GmALMT23*, Glyma.12G094400.1.p), *Malus domestica* (*MdALMT7*, MD03G1155500), *Vitis vinifera* (*VviALMT2*, VIT_206s0009g00450.1), *Citrus clementina* (*CcALMT7*, Ciclev10030132m), *Theobroma cacao* (*TcALMT7*, Thecc.09G272800.1.p), *Gossypium hirsutum* (*GhALMT7*, Gohir.D09G098400.1.p), *Zea mays* (ZmALMT7, Zm00001d025373_P001), *Sorghum bicolor* (*SbALMT7*, Sobic.006G075200.1.p), *Musa acuminata* (*MaALMT7*, GSMUA_Achr8P30390_001). Phylogenetic analysis of *ALMT7* homologs from different species and *AtALMT1* revealed a clade containing the *Arabidopsis* and *Brassica ALMT7*s, with *AtALMT1* sister to the *ALMT7*s (**Figure 1A**), confirming *BnALMT7-A4* and *BnALMT7-C4* as most closely related to *AtALMT7*. These proteins are part of a larger group of related dicot proteins, with the monocot closest relatives lying in a separate clade (**Figure 1A**).

Aluminum-activated malate transporters (ALMTs) typically are consist of 5–7 transmembrane domains (TMDs) and a cytosolic domain (CTD). To predict TMDs and protein structures, we employed both the deepTMHMM and Alphafold3 servers for BnALMT7s (**Figures 1B, C**). Our alignment analysis revealed that BnALMT7s contain six conserved TMDs at the N terminus and a highly aligned C-terminal domain (**Figure 1D**). The conserved TMDs are essential for protein’s membrane localization and malate transport, which form core structure of malate channel. The alignment analysis also revealed that *At*ALMT7 and *Bn*ALMT7-A4 shared a more conserved C terminus (74.54% identity) than *Bn*ALMT7-C4 and *At*ALMT7 (69.37% identity). Our findings support and extend those of Dabravolski and Isayenkov (2023). Specifically, BnALMT7-A4 and AtALMT7 exhibit a unique 26 amino acids following the sixth TMD that are not present in BnALMT7-C4 or other plant relatives (**Figure 1D**). These results suggest that *BnALMT7-A4* was the closest homolog of *AtALMT7* in rapeseed.

### Expression of *BnALMT7*s in response to Al stress

**Figure 2:**
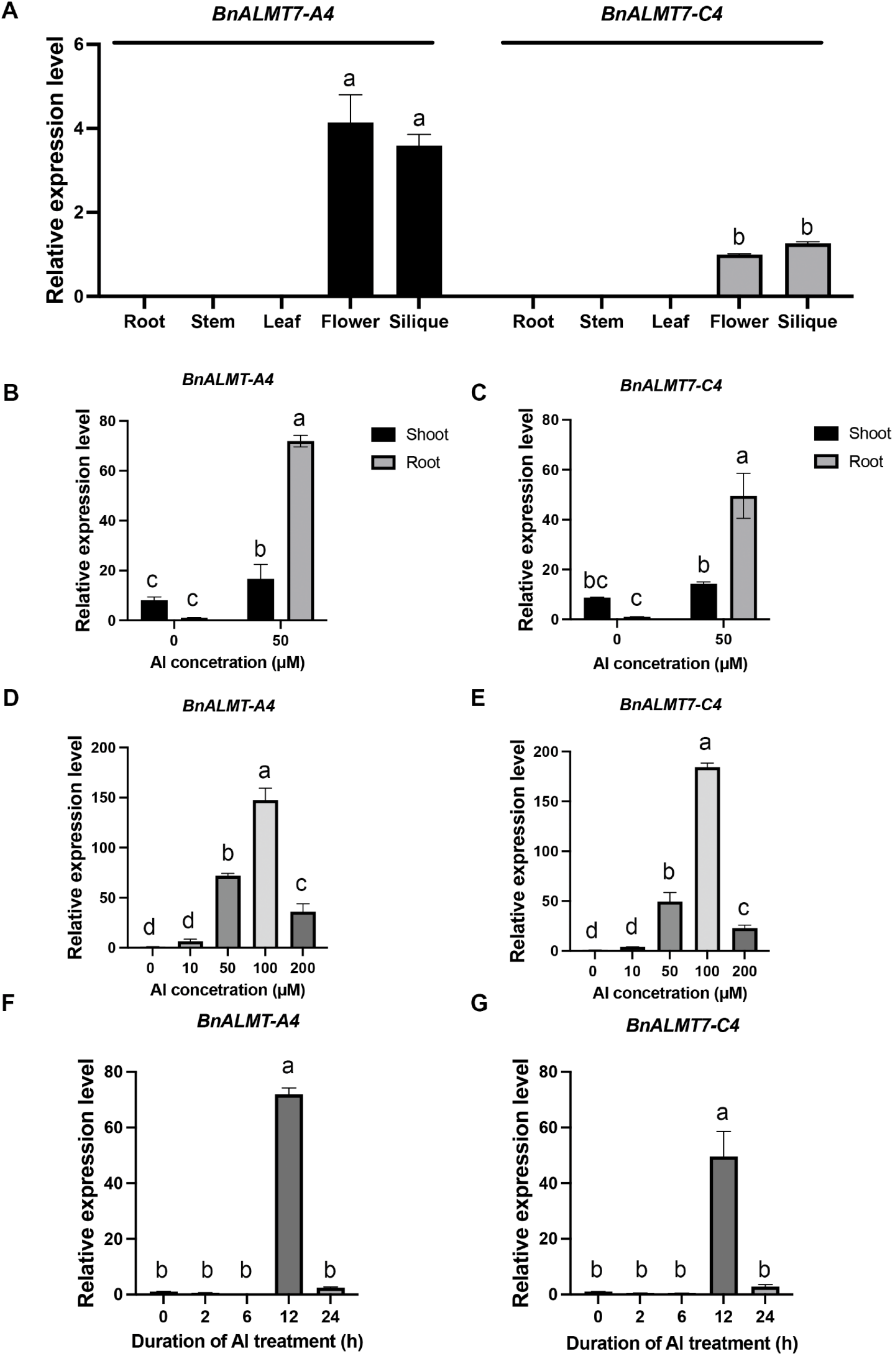
Expression analysis of BnALMT7-C4 and BnALMT7-A4 in rapeseed seedlings. **(A)** Expression patterns of BnALMT7s in rapeseed. **(B, C)** Expression of BnALMT7s in shoots and roots. Seedlings were exposed to 0.5mM CaCl_2_ solution (pH 4.5) containing 50µM AlCl_3_ for 12 h. Expression of BnALMT7s in shoots and roots were determined, respectively. **(D, E)** Dose-response expression of BnALMT7s in rapeseed roots. Seedlings were exposed to 0.5mM CaCl_2_ solution (pH 4.5) containing 0, 10, 50, 100 and 200µM AlCl_3_ for 12h. **(F, G)** Time-dependent expression of BnALMT7s in rapeseed roots. Seedling were exposed to 0.5mM CaCl_2_ solution (pH 4.5) containing 50µM AlCl_3_ for 0, 2, 6, 12, 24 h. Mean values ± SD of three biological replicates are given. Different uppercase letters indicate significantly different means (P<0.05, ANOVA test followed by Tukey test).

We performed quantitative reverse transcription PCR (qRT-PCR) analysis to evaluate the expression patterns of *BnALMT7-A4* and *BnALMT7-C4* in different tissues of soil-grown *Brassica napus* L. cultivar ‘ZS11’. Under normal growth conditions, the transcripts of both *BnALMT7*s were present in flowers and siliques, while no transcript was detected in other tissues (roots, leaves and stems) (**Figure 2A**). However, in hydroponic cultured seedlings, Al treatment significantly enhanced expression of *BnALMT7-A4* in roots and shoots, while a significant increase of *BnALMT7-C4* expression was observed only in roots (**Figures 2B, C**). The Al-induced increase in *BnALMT7-A4* was considerably larger in roots (**Figures 2B, C**). Dose-response and time-course experiments revealed that expression of *BnALMT7*s in roots was induced by Al treatment in a concentration-dependent manner and a time-dependent manner. Transcript levels of both genes were most abundant at 100 µM AlCl_3_ (**Figures 2D, E**) and 12 h post-induction (**Figures 2F, G**) respectively. Taken together, these data show that both *Bn*ALMT7s are highly Al-inducible in roots but may have additional functions unrelated to Al in the flowers and siliques.

### Localisation and homodimerization of *Bn*ALMT7-A4

**Figure 3:**
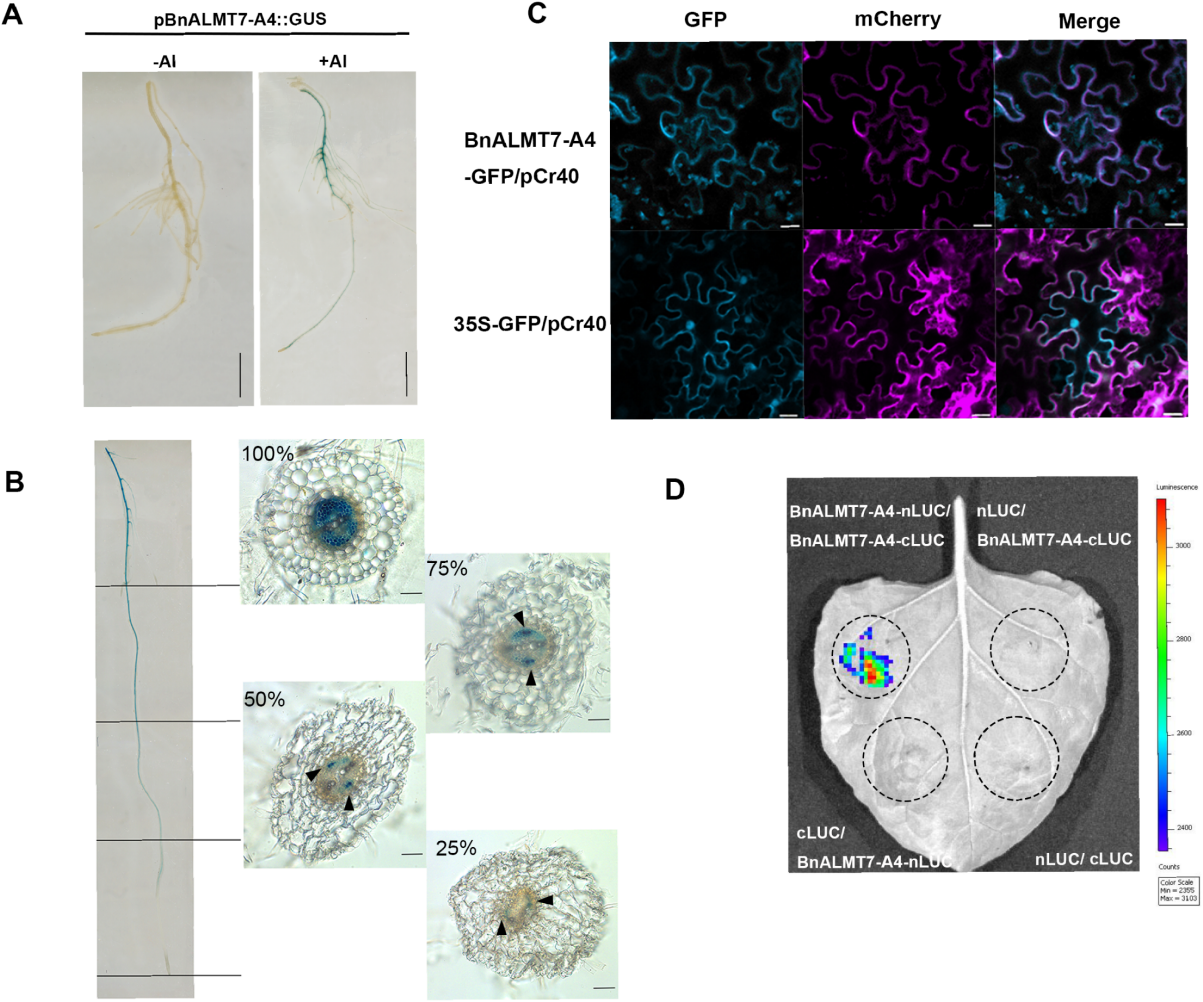
Localization and self-interaction of BnALMT7-A4. **(A)** GUS staining snalysis of rapeseed hairy roots expressing pBnALMT7-4A::GUS with or without AlCl_3_ treatment. **(B)** Cross sections of GUS stained rapeseed hairy root expressing pBnALMT7-A4::GUS with AlCl_3_ treatment. Root was segmented into 4 even parts, including 25% from root tip, 25% to 50% from root tip, 50% to 75% and 75% and above part of root, respectively. The cross sections were performed in each part, separately, with scale bar = 50 µm. Black triangles indicate phloem **(C)** Subcellular localization of BnALMT7-A4 in tobacco leaves, and GFP-tagged empty vector was used as control, with scale bar = 20 µm. **(D)** Split luciferase complementation assay to detect self-interaction of BnALMT7-A4.

*BnALMT7-A4* was cloned for functional studies. To further understand the expression pattern of *BnALMT7-A4* in rapeseed, we generated *pBnALMT7-A4*::GUS transgenic rapeseed hairy root lines. GUS staining showed that the *BnALMT7-A4* promoter was highly induced by Al treatment in roots, particularly in the vascular system (**Figures 3A, B**). Cross-section analysis showed that the *BnALMT7-A4* promoter was mainly expressed in the stele in the mature zone and more specifically in the phloem in the elongation zone and meristem, while activity of *pBnALMT7-A4::GUS* gradually decreased along roots longitudinally towards the root tip (**Figure 3B**). The subcellular localization of *Bn*ALMT7-A4 was determined by transient expression of *35S*::*BnALMT7-A4*-GFP in tobacco (*Nicotiana benthamiana*) leaves (**Figure 3C**). The green fluorescence of *Bn*ALMT7-A4-GFP was found to overlap considerably with the red fluorescence of the plasma membrane marker pCr40 (PIP2A-mCherry), while the *35S*::GFP control was found in both nuclei and the cytosol, indicating that *Bn*ALMT7-4A was a plasma membrane-localized protein (**Figure 3C**). As other ALMTs can form homodimers and the Alphafold 3.0 server predicted that *Bn*ALMT7-A4 could form homodimers, we tested the ability of *Bn*ALMT7-A4 to form homodimers via a split-luciferase assay in tobacco leaves (**Figure 3D**, **Supplementary Figure 2**). A strong luciferase signal was observed, due to the self-interaction of *Bn*ALMT7-A4 proteins, and no signal was detected in the negative controls. These findings suggest that plasma membrane-localized protein *Bn*ALMT7-A4 could interact with itself.

### Overexpression of *BnALMT7-A4* enhanced Al tolerance in *Arabidopsis*

**Figure 4:**
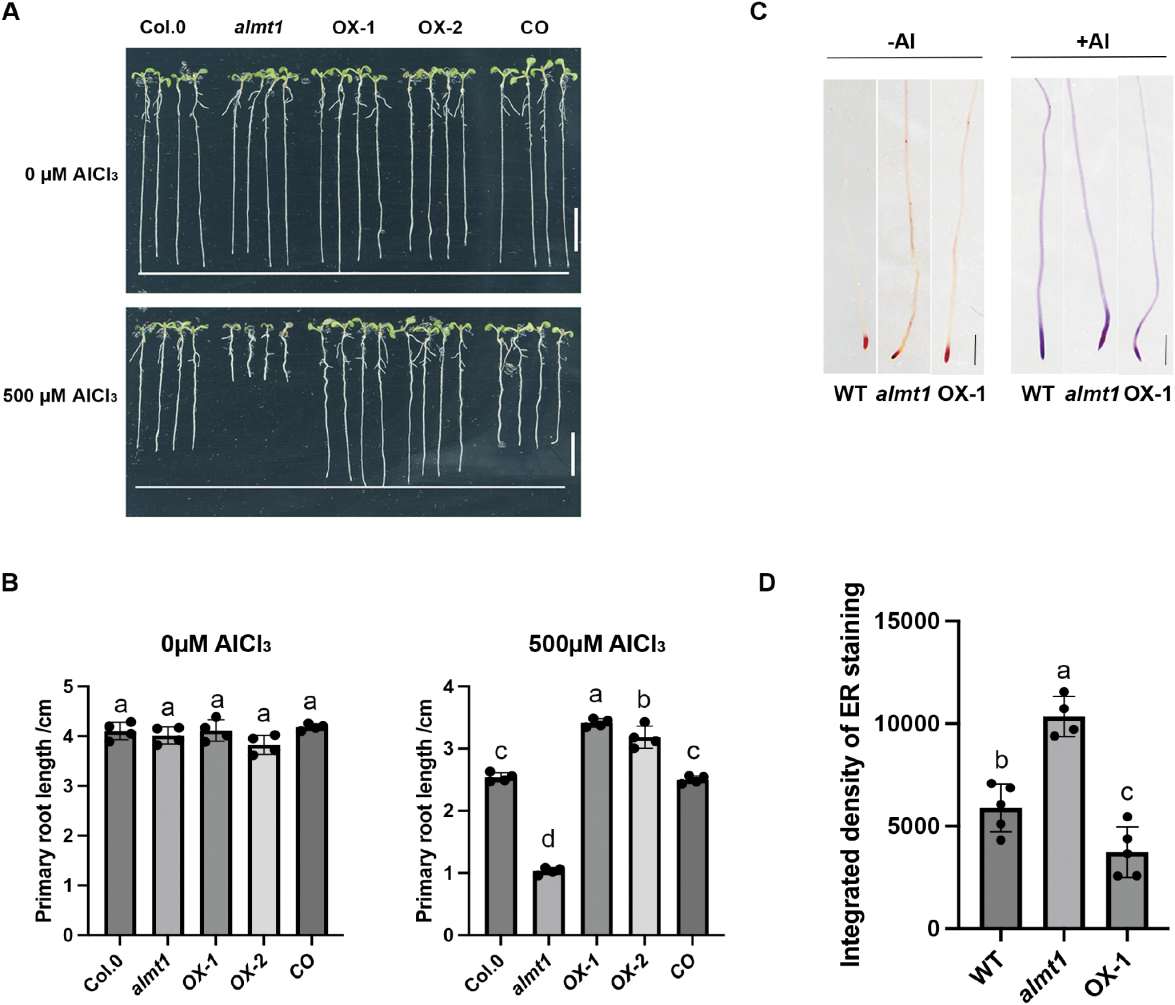
Overexpression of BnALMT7-4A in Arabidopsis resulted in enhanced Al tolerance. **(A, B)** Representative photographs **(A)** and quantitative data **(B)** of Col.0, almt1, two BnALMT7-A4 overexpression lines (OX-1 and OX-2) and heterologous complementation of BnALMT7-A4 in almt1 (CO) under absence or presence of 500 µM AlCl_3_ (n=4), with scale bar= 1cm. **(C, D)** Effect of BnALMT7-4A overexpression on Al accumulation in root tips (n=4), with scale bar = 100 µm. Different uppercase letters indicate significantly different means (P<0.05, ANOVA test followed by Tukey test).

To investigate the function of *BnALMT7-A4* in response to Al stress, we firstly generated two independent transgenic *Arabidopsis* lines overexpressing *BnALMT7-A4* (OX-1 and OX-2) in wild type and a ‘cross-complementation’ line overexpressing *BnALMT7-A4* in the *Atalmt1* mutant (Hoekenga et al., 2006) (**Supplementary Figure 3**). Al tolerance of the different *Arabidopsis* lines was determined by measuring primary root length in different growth conditions. All lines showed a similar root length without Al stress (**Figures 4A, B**). When treated with 500µM AlCl_3_ the *Atalmt1* mutant showed a greatly shortened root length compared to wild type as previously reported (Hoekenga et al., 2006). The two lines overexpressing *BnALMT7-A4* in wild-type *Arabidopsis* possessed significantly longer primary roots under Al stress compared to WT (**Figures 4A, B**), showing that *Bn*ALMT7-A4 can enhance Al tolerance in wild-type *Arabidopsis*. The ‘cross-complementation’ line behaved similarly to wild-type under Al treatment demonstrating that *BnALMT7-A4* could replace the function of *AtALMT1* under Al stress (**Figures 4A, B**). To compare Al accumulation in root tips of wild type, *Atalmt1* and *BnALMT7-A4-OX1*, ECR staining was used. We found that the *Atalmt1* mutant showed significantly enhanced Al accumulation in root tips when compared with WT under no-Al conditions, while decreased Al accumulation was found in the overexpression line of *BnALMT7-A4* under Al stress (**Figures 4C, D**). These results suggested that heterologous expression of *BnALMT7-A4* positively regulates Al tolerance in both wild type and *Atalmt1* mutant *Arabidopsis*.

### Overexpression of *BnALMT7-A4* enhanced Al tolerance in rapeseed hairy roots

**Figure 5:**
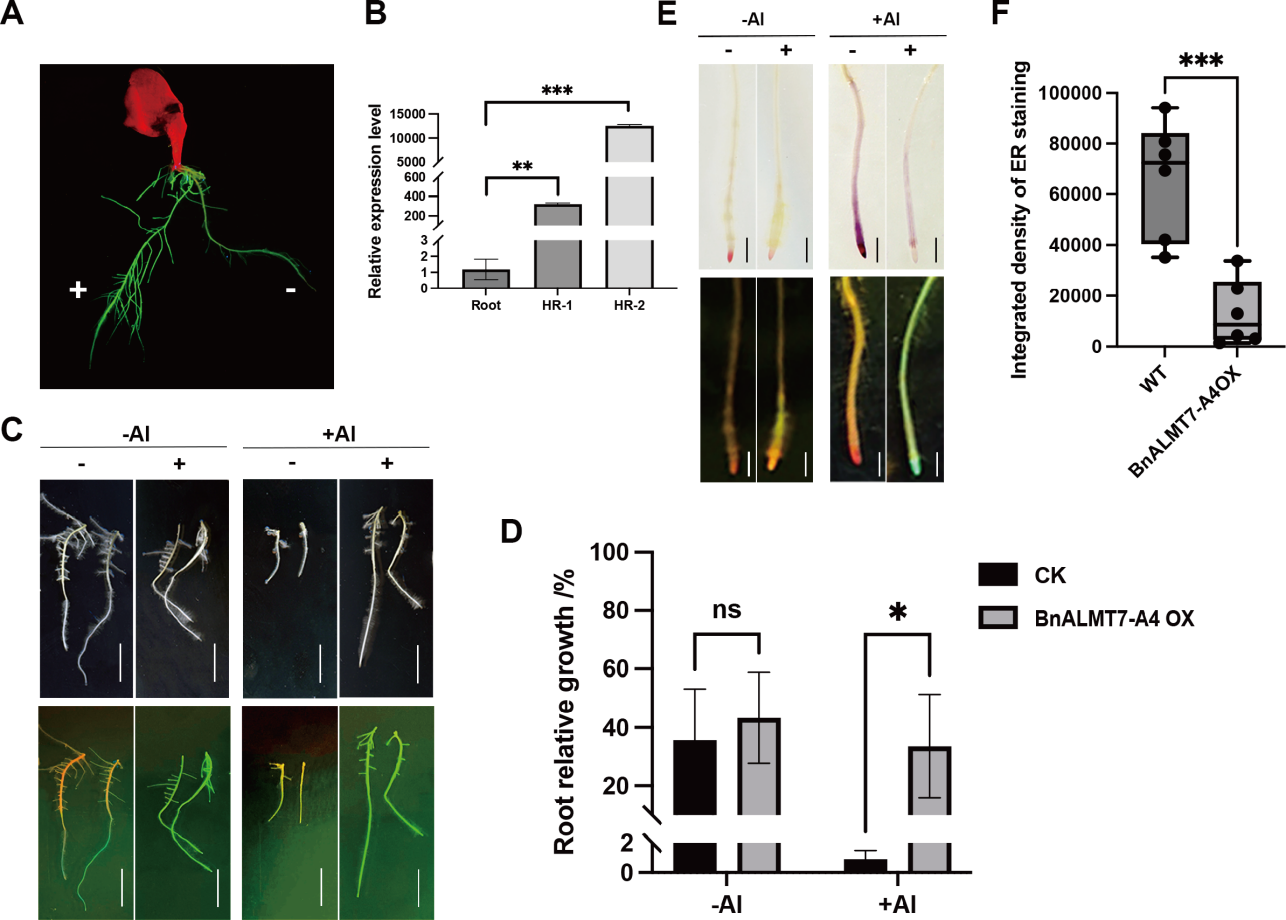
Overexpression of BnALMT7-4A by using *Agrobacterium rhizogenes* mediated hairy root transformation in rapeseed. **(A)** The hairy roots from cotyledons of rapeseeds. Roots with green fluorescence were marked with addition sign (+) indicated transformed hairy roots, and roots without green fluorescence marked with minus sign indicated untransformed hairy roots. **(B)** Expression quantification of BnALMT7-A4 in untransformed (Root) and two independent transformed hairy roots (HR-1 and HR-2). **(C, D)** Effect of BnALMT7-4A overexpression on Al accumulation in root tips (n=6), with scale bar = 100 µm. **(E, F)** Representative photographs **(E)** and quantitative data **(F)** of WT and BnALMT7-A4 overexpression hairy roots under absence or presence of 50µM AlCl_3_ (n=3-4), with scale bar= 1cm. Asterisks indicate statistically different values (Šídák’s multiple comparsions test, *P < 0.05, **P < 0.01, ***P < 0.001).

To further study the function of *BnALMT7-A4* upon Al stress, rapeseed hairy root transformation was carried out to generate overexpression hairy root lines in *Brassica napus* cultivar ‘ZS11’. Transformed hairy roots were visualized by expression of green fluorescent protein (GFP) (**Figure 5A**). The expression of *BnALMT7-A4* in transformed roots was then determined using qRT-PCR, and the expression of *BnALMT7-A4* in untransformed hairy roots was measured as the control (**Figure 5B**). Phenotypic analysis showed that overexpression lines of *BnALMT7-A4* had significantly increased root elongation upon Al treatment compared with untransformed lines (**Figures 5C, D**). No significant difference on root elongation was observed between transformed and untransformed hairy roots without Al stress (**Figures 5C, D**). In addition, we found significantly reduced Al accumulation in root tips of *BnALMT7-A4* overexpressing lines compared to untransformed hairy roots under Al stress (**Figures 5E, F**). Taken together, these results suggest that overexpression of *BnALMT7-A4* enhanced the Al tolerance in rapeseed hairy roots.

### RNA-seq analysis of *BnALMT7-A4* overexpressing hairy roots in response to Al stress

**Figure 6:**
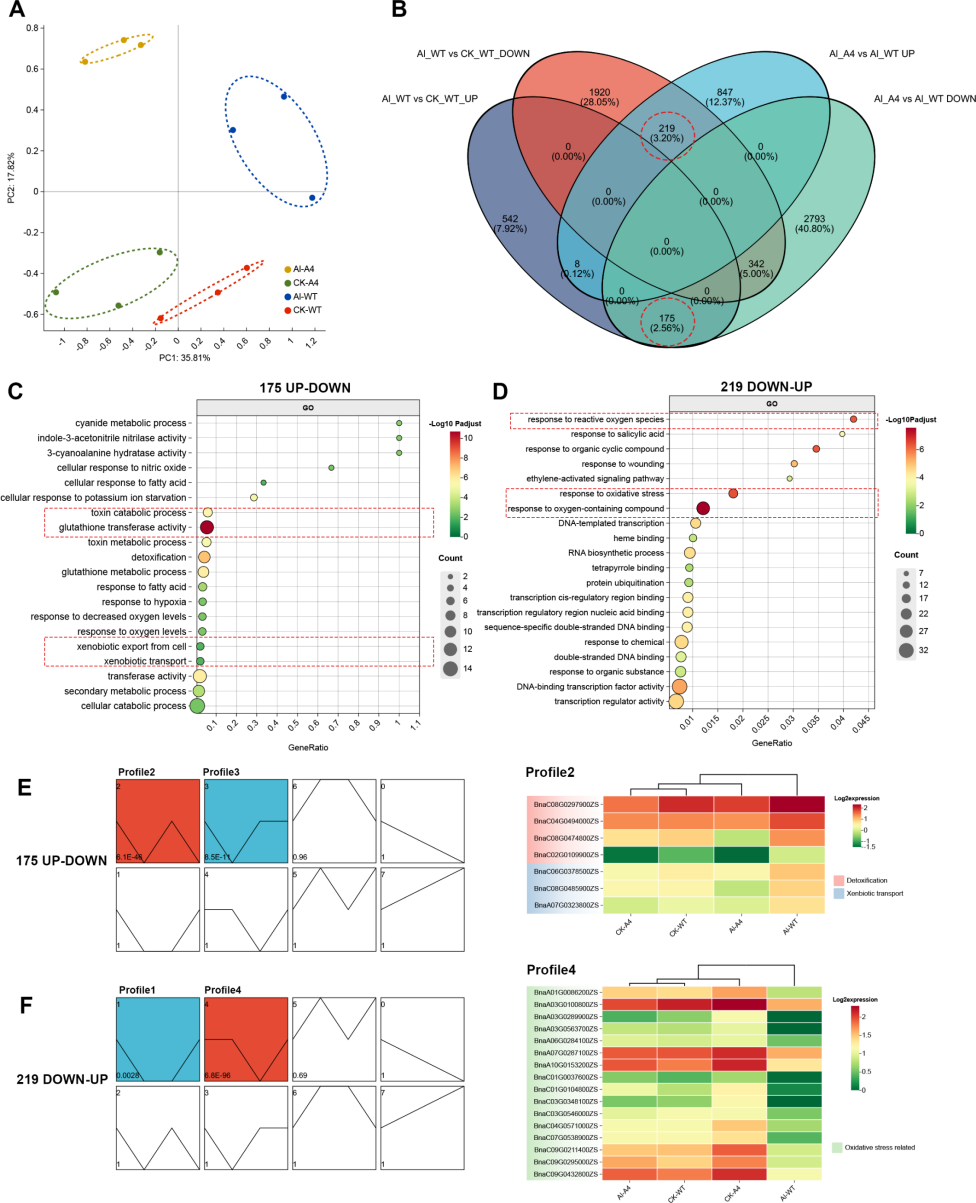
Transcriptomic analysis of rapeseed hairy roots overexpressing BnALMT7-A4 with or without AlCl_3_ treatment. **(A)** Principle component analysis (PCA) of the RNA-seq samples. **(B)** Venn Diagram of up-and down-regulated DEGs from two pairwise comparisons (Al-WT vs CK-WT and Al-A4 vs Al-WT) to identify DEGs with reversed expression patterns between two comparisons. **(C, D)** Gene ontology (GO) enrichment analysis of top 20 GO terms of DEGs, upregulated in Al-WT vs CK-WT **(C)** and upregulated in Al-A4 vs Al-WT **(D)**. **(E)** Expression trend analysis of 175 DEGs from GO term enrichment analysis (left panel). The expression data of Al-responsive genes from profile2 (right panel). Log2 (TPM) values were used for heatmap construction. **(F)** Expression trend analysis of 219 DEGs from GO term enrichment analysis (left panel). The expression data of DEGs from profile4 (right panel). Log2 (TPM) values were used for heatmap construction.

We performed transcriptomic sequencing using overexpressing hairy roots (*BnALMT7-A4*OX) and untransformed hairy roots (WT) with (Al) or without (CK) Al treatment (each with 3 biological replicates). Principal component analysis (PCA) showed that the 3 biological replicates of each condition were significantly clustered, indicating the reliability of the sequencing results (**Figure 6A**). Principal component 1 (PC1), which accounts for 35.81% of the total variation, effectively differentiated *BnALMT7-A4*OX from wild-type (WT) hairy roots. Principal component 2 (PC2), representing 18.82% of the total variation, was able to distinguish between samples subjected to Al treatment and those that were not treated (**Figure 6A**).

A total of 732 differentially expressed genes (DEGs) were identified in the comparison between *BnALMT7-A4*OX and WT hairy roots without Al treatment (CK_A4 vs CK_WT) with a threshold of |log2| (relative expression level) ≥ 1 and padj ≤ 0.05. Additionally, 3,206 DEGs were found in the WT hairy roots, comparing samples with and without aluminum treatment (Al_WT vs CK_WT), while 7,648 DEGs were identified in the *BnALMT7-A4*OX hairy roots under the same conditions (Al_A4 vs CK_A4) (**Supplementary Figure 4**). Our previous data suggested that *Bn*ALMT7-A4 enhances tolerance to Al stress. Thus, we looked for overlapping DEGs from “Al_WT vs CK_WT” and “Al_WT vs Al_A4” to identify potential Al-responsive genes, expression of which was affected in the opposite direction by *BnALMT7-A4* overexpression under Al stress. To obtain DEGs with reversed expression patterns, genes were classified into upregulation and downregulation groups (Al_WT vs CK_WT Up, Al_WT vs CK_WT Down, Al_WT vs Al_A4 Up and Al_WT vs Al_A4 Down). A Venn diagram of these four groups was constructed and showed that 175 DEGs were upregulated in Al_WT vs CK_WT but downregulated in Al_WT vs Al_A4 (named 175-UP-DOWN), while 219 DEGs were downregulated in Al_WT vs CK_WT, and upregulated in Al_WT vs Al_A4 (named 219-DOWN-UP) (**Figure 6B**).

GO enrichment analysis of 175-UP-DOWN and 219-DOWN-UP was performed, and the top 20 GO terms were shown in **Figures 6 C, D**, respectively. The 175-UP-DOWN genes that were upregulated by Al stress in untransformed roots but downregulated in the presence of *Bn*ALMT7-A4 under Al stress showed GO-enrichment of terms related to detoxification, such as ‘glutathione transferase activity’, ‘toxin catabolic process’, ‘xenbiotic transport’. This might suggest that overexpression of *BnALMT7-A4* likely alleviated Al toxicity in hairy roots, resulting in reduced expression of other detoxification related genes (**Figure 6C**). GO analysis of the 219-DOWN-UP genes that were downregulated by Al stress in control plants but upregulated in the presence of *Bn*ALMT7-A4 under Al stress showed enrichment of the terms ‘response to oxygen-containing compound’, ‘response to oxidative stress’ and ‘response to reactive oxygen species’ (**Figure 6D**). We hypothesized that that expression of more oxidative stress-responsive genes was increased in *BnALMT7-A4* overexpressing hairy roots to help plants cope with the oxidative stress caused by Al treatment. KEGG pathway analysis showed that the 175-UP-DOWN genes were mainly enriched in ‘Glutathione metabolism’, ‘Tyrosine metabolism’, ‘Starch and sucrose metabolism’ pathways, while the 219-DOWN-UP genes were significantly enriched in pathways including ‘Nitrogen metabolism’, ‘Arginine and proline metabolism’ and ‘Cysteine and methionine metabolism’ (**Supplementary Figure 5**).

The expression data from the 175-UP-DOWN genes and the 219-DOWN-UP genes were further enriched by using expression trend analysis, where genes with similar expression trends across genotypes and treatments were clustered. Two significantly clustered trend profiles were identified in each of 175-UP-DOWN and 219-DOWN-UP, respectively (**Figures 6E, F**). In 175UP-DOWN, profile 2 (97 genes, p = 6.1e^-46^) and profile 3 (39 genes, p = 8.5 e^-11^) were identified (**Figure 6E**). 219-DOWN-UP genes were significantly clustered into profile 1 (12 genes, p=0.0028) and profile 4 (191, p=6.8e^-96^) (**Figure 6F**). As shown in **Figure 6E**, expression of genes in profile 2 was significantly upregulated in Al-treated untransformed roots compared to untreated controls and down-regulated in all plants overexpressing *Bn*ALMT7-A4 regardless of Al stress status. Profile 4 from 219-DOWN-UP showed genes that were down-regulated by Al stress in both untransformed roots and *Bn*ALMT7-A4-overexpressing roots but were not downregulated by Al stress in untransformed roots (**Figure 6**). Similar to the previous GO term analysis (**Figures 6C, D**), the genes of profile 2 were mainly enriched in ‘detoxification’ and ‘xenbiotic transport’ terms and profile 4 genes were enriched in ‘oxidative stress related’ terms: a heatmap of the associated genes in each GO category is shown in **Figures 6E, F**. For profile 2, detoxification efflux carriers *DETOXIFICATION 12* and *14* (*DTX12* and *DTX14*, BnaC08G0485900ZS and BnaA07G0323800ZS) were significantly upregulated in both Al-WT vs CK-WT and Al-WT vs CK-A4 with fold-change value from 2.2 to 5.4. However, in Al-A4 vs Al-WT comparison, expression of those genes were significantly downregulated by 70 to 90%, which led to no significant difference in Al-A4 vs CK-A4. Similar expression pattern was also identified in other detoxification or xenobiotic transport-related genes, *GLUTATHIONE S-TRANSFERASE TAU* (*GSTU4/5/25*, BnaC04G0494000ZS/BnaC04G0185400ZS/BnaC08G0474800ZS) with their downregulation fold-change value from 0.03 to 0.36 in Al-A4 vs Al-WT. In profile 4, the potential peroxidase *CATALATASE 2* (CAT2, BnaC01G0037600ZS) and ethylene synthesis related gene *1-AMINOCYCLOPROPANE-1-CARBOXYLATE SYNTHASE 6* (*ACS6*, BnaC09G0295000ZS) was found upregulated in Al-A4 vs Al-WT comparison with fold-change values of 2.9 and 5.5, respectively. The similar expression trend was found in oxidative stress responsive transcription factors, such as *ETHYLENE-RESPONSIVE TRANSCRIPTION FACTOR 6* (*ERF6*, BnaC01G104800ZS with 8.1 fold upregulation), *BTB/POZ DOMAIN CONTAINING PROTEIN 5* (*BTBD5*, BnaA030563700ZS, with 7.1 fold upregulation) and *ZINC FINGER OF ARABIDOPSIS THALIANA 12* (*ZAT12*, BnaC09G0432800ZS with 6.1 fold upregulation).

## Discussion

In previous studies, a total of 39 *BnALMT* family members were identified, corresponding to 14 publicly reported *AtALMT* members in *Arabidopsis* (Din et al., 2021). *ALMT* family members have been identified in many other species, including 21 in apple (*Malus domestica*) (Linlin et al., 2018), 34 in soybean (*Glycine max*) (Peng et al., 2018), 9 in rice (*Oryza sativa*) (Delhaize et al., 2007), 17 in rubber tree (*Hevea brasiliensis*) and 24 in poplar (*Populus trichocarpa*) (Ma et al., 2020). In our study, two *BnALMT7*s were identified as the closest homologs of *AtALMT7* by BLAST and phylogenetic analysis with *AtALMT1*, *AtALMT7* and *ALMT7* homologs of other species (**Figure 1A**). Two *BnALMT7*s were closely clustered with *AtALMT7* in the same clade as *AtALMT1* (**Supplementary Figure 1**). These results suggest that two homologs of *AtALMT7* were identified in rapseed, *BnALMT7-A4* and *BnALMT7-C4*. However only one homolog, *Bn*ALMT7-A4, possessed the 26 amino acid insertion in the C-terminal domain also seen in *At*ALMT7 (Dabravolski and Isayenkov, 2023). Although the biological function of this insertion is unknown, it would be interesting to investigate its functional role, considering the insertion is located between GABA binding motif and key Al sensing residues (Liu and Zhou, 2018; Dabravolski and Isayenkov, 2023).

The structure of proteins contributes to their functions and activities. Cryoelectron microscopy (cryo-EM) structure analysis revealed *At*ALMT1 anion channel structures and the key amino acids for malate recognition and transport in different conditions (Wang et al., 2022). Recent work resolved the vacuolar chloride channel structure of *At*ALMT9 for stomata aperture regulation in *Arabidopsis*. The structures of *Gm*ALMT12 and *At*ALMT9 anion channels existing as homodimers have also been resolved, which extended knowledge of their function on stomata aperture regulation (Qin et al., 2022; Qian et al., 2024). The tertiary structures of *Bn*ALMT7-A4 and *Bn*ALMT7-C4 were predicted and analyzed by using the Alphafold server, which provided structural insight on their functions and regulatory mechanisms (**Figure 1C**). Transmembrane domain prediction showed that both *Bn*ALMT7s contained six predicted TMDs (**Figures 1B, C**). The C-terminal domains (CTDs) of ALMTs also play a crucial role in their function. The CTD of *At*ALMT1 is involved in adjusting its own homodimerization, and a conserved CTD of Ma1G (ALMT9) in apple was reported to affect its malate transport activity (Li et al., 2020a; Wang et al., 2022). In our studies, previously reported key residues were found in C terminus of both *Bn*ALMT7s (e.g. Lys-425, Phe-424 and Leu-418 in *Bn*ALMT7-A4): mutation of these residues may disrupt dimeric assembly (Li et al., 2020a). Furthermore, self-interaction of *Bn*ALMT7-A4 was confirmed by using a spilt-luciferase complementation assay (**Figure 3C**), which is consistent with the homodimeric structures reported for *At*ALMT1, *At*ALMT9, *Os*ALMT7 and *Gm*ALMT12 (Qin et al., 2022; Wang et al., 2022; Zhou et al., 2022; Qian et al., 2024). Recent studies also showed that some ALMTs could work as heterodimers, such as Ma1α and Ma1β in apple (Li et al., 2024). In the future, high-resolution ALMT structures in dimeric forms would further our knowledge on the function and regulation mechanisms of ALMT proteins.

The *Arabidopsis* Al tolerance protein *At*ALMT1 is enriched in the root epidermis and induced in root tips by Al, regulating malate release from roots (Hoekenga et al., 2006; Kobayashi et al., 2007). *SlALMT4* and *SlALMT5* had been reported to express in vascular bundles in fruit at the mature green stage and in embryos in mature seeds; overexpression of *SlALMT5* increased both malate and citrate content in mature seeds (Sasaki et al., 2016). The first identified ALMT, *Ta*ALMT1 acted as a transporter for both malate and gamma-aminobutyric acid (GABA), which may confer Al^3+^ and alkaline pH tolerance in barley, respectively (Kamran et al., 2020). In soil-grown, unstressed rapeseed, the transcripts of both *BnALMT7*s were only detected in flowers and siliques (**Figure 2E**), which was consistent with previous analysis of *BnALMT7*s and expression of *AtALMT7* from *Arabidopsis* (Klepikova et al., 2016; Din et al., 2021). Addition of Al induced *BnALMT7*s expression to a larger extent in roots than in shoots (**Figure 2**) and the activity of *pBnALMT7-A4::GUS* was induced in the stele of mature root zone (**Figure 3B**). The GUS signal gradually became restricted to the phloem cells travelling along the root from the mature zone towards the root tips (**Figure 3B**). Phloem cells are involved in translocation of shoot-derived chemicals and proteins to the roots, including sugar, auxin, organic acids (Dinant and Lemoine, 2010; Agustí and Blázquez, 2020). Thus, *BnALMT7-A4* represented a good candidate for manipulating plant Al tolerance and speculated that *Bn*ALMT7-A4 may be involved in the long-distance transport of malate or other organic or inorganic anions from the shoot to the root.

In an attempt to generate Al-tolerant plants, *BnALMT7-A4* was overexpressed in *Arabidopsis* and rapeseed hairy roots subject to Al stress (**Figures 4**, **5**). In *Arabidopsis*, the *BnALMT7-A4* overexpression lines exhibited longer root elongation and reduced Al accumulation in root tips under Al treatment (**Figure 4**). Similar phenotypes under Al treatment were observed in transgenic rapeseed hairy roots overexpressing *BnALMT7-A4* (**Figure 5**). These results demonstrated that overexpression of *BnALMT7-A4* decreased Al absorption in roots and enhanced Al tolerance in both *Arabidopsis* and rapeseed hairy roots, which was similar to other *ALMT* homologs, including *AtALMT1* in Arabidopsis, *GmALMT1* in soybean, *MsALMT1* in alfalfa, *BnALMT1* and *BnALMT2* in rapeseed and *BoALMT1* in cabbage (Ligaba et al., 2006; Kobayashi et al., 2007; Liang et al., 2013; Zhang et al., 2018; Jin et al., 2024).

To understand the mechanisms by which *BnALMT7-A4* confers Al tolerance, we carried out transcriptomic analysis of rapeseed hairy roots overexpressing *BnALMT7-A4* treated with or without Al. GO term analysis of 732 DEGs from CK-4A vs CK-WT was applied to study effect of *BnALMT7-A4* overexpression in *Brassica napus*. The expression of both *MALATE DEHYDROGENASE 1* and *2* were significantly upregulated, which could be induced by overproduction of malate in the overexpression lines. Abiotic stress related GO terms like “response to abscisic acid” and “response to salt stress” were enriched containing *CYP707A* and *OPEN STOMATA 1* (*OST1)* etc., indicating the overexpression of *BnALMT7-A4* may preactivated the plant defense system to cope with the potential stresses. *CYP707A* was highly upregulated in the overexpression lines, which was reported to response to salt, osmotic, dehydration stresses and ABA in *Arabidopsis {Citation}*. Mustilli et al. reported that Arabidopsis OST1, expressed in guard cells and vascular tissues, was triggered by ABA in regulating stomatal closure and played a role in ROS production (Mustilli et al., 2002; Kushiro et al., 2004). Another study showed that *OST1* responsed to low phosphorous via transcriptionally activated by PHOSPHATE STARVATION RESPONSE 1 (PHR1), which regulated tomato root system architecture (Liu et al., 2025). For down-regulated DEGs, “cell wall organization”, “cellulose biosynthesis process” terms were most significantly enriched, which implied that overexpression of *BnALMT7-A4* decreased root cell wall dynamic modification. More “oxidoreductase activity” and “detection of oxygen” terms enriched indicated that lower level of ROS and reallocation of in overexpression lines. *PEROXIDASE* family members (*PER1*/*34*/*62*/*66*) were enriched, which participated in maintaining ROS homeostasis in plant cells and displayed multifunctions in plant development and stress response. In *Arabidopsis*, AtPER34 was reported to play a role in H_2_O_2_ generation during defense response, while other PERs could decompose H_2_O_2_ in different ways (O’Brien et al., 2012). AtPER1 together with AtPER44 and AtPER73 were reported to control cell wall properties by maintaining ROS homeostasis during polar expansion of root hair cell (Marzol et al., 2022). Wu et al. found that overexpression of *AtPER64* increased Al tolerance in tobacco (Wu et al., 2017).

Further analysis revealed two significantly clustered profiles of expression trends (**Figure 6**). Profile 2 represented genes upregulated in WT by Al treatment that were downregulated in *BnALMT7-A4* overexpression line treated with Al. Profile 4 contained genes with the reversed expression trend (**Figures 6E, F**). Combining the GO term enrichment analysis with trend analysis, we found that genes of Profile 2 were associated with detoxification and xenbiotic transport, and genes in Profile 4 were associated with response to Reactive Oxygen Species (ROS) and response to oxidative stress (**Figures 6C, D**). GSTU family members from Profile 2 are involved in degradation of H_2_O_2_ and xenbiotic detoxification in Arabidopsis (Gunning et al., 2014; Santamaría et al., 2018). MATE family members functioned as detoxifying efflux carriers for antibiotics and toxic compounds (Li et al., 2002; Miyauchi et al., 2017). In profile 4, ZING FINGER PROTEIN 12 (ZAT12) and ETHYLENE RESPONSE FACTOR 6 (ERF6) played crucial roles in reactive oxygen signaling and cold stress responses in *Arabidopsis* (Davletova et al., 2005; Dubois et al., 2015; Xu et al., 2021). *CATALASE 2* (*CAT2*) encodes a peroxisomal catalase, which was necessary for redox homeostasis in *Arabidopsis* (Vanderauwera et al., 2005; Bueso et al., 2007; Queval et al., 2007).

Plant hormones serve as signaling compounds involved in plant growth and developmental processes and stress response regulations (Waadt et al., 2022; Zhu et al., 2024). ‘response to salicylic acid’ was enriched in group-219-DOWN-UP, and ‘response to jasmonic acid’ and ‘ethylene receptor activity’ were enriched in group-175-UP-DOWN. Exogenous methyl jasmonate treatment was found to improve antioxidant performance in blueberry (*Vaccinium corymbosum*) (Ulloa-Inostroza et al., 2017). KEGG enrichment analysis of DEGs in group-175-UP-DOWN and group-219-DOWN-UP showed enriched metabolic pathways for 11 amino acids (proline, arginine, methionine, cysteine, glycine, serine, threonine, lysine, aspartate, glutamate, tryptophan) and the tripeptide glutathione (composed of cysteine, glutamate, and glycine), which indicated that overexpression of *BnALMT7-A4* increased the metabolism of specific amino acids (**Supplementary Figure 5**). These amino acids may directly function as antioxidants, involved in oxidative stress responses. For example, proline was reported to decrease salt-induced oxidative stress by increasing the activity of antioxidants and decreasing ROS content in wheat, sorghum and maize (Zulfiqar and Ashraf, 2023). In addition, the reduced form of glutathione is an antioxidant that functions in ROS scavenging and cellular redox balance (Noctor et al., 2024). Recent studies also demonstrated that exogenous glutathione application not only promoted elimination of H_2_O_2_ and peroxides in rice roots, it also enhanced biosynthesis of phytochelatin, which played important roles in Al3+ sequestration in vacuole (Jiang et al., 2025). Thus, the changes in amino acid metabolism may be part of larger metabolic shifts associated with Al stress.

Al triggered a series of phytotoxic effects in plants, such as impairment of root growth and development, a decline in photosynthetic capacity and overall plant growth, increased accumulation of ROS and the subsequent damage to cellular and biochemical components (Ofoe et al., 2023). The downregulation and upregulation of corresponding genes in *BnALMT7-A4* overexpression lines compared with WT under Al treatment revealed that overexpression of *BnALMT7-A4* enhanced Al tolerance in rapeseed hairy roots by enhancing their ability in response to oxidative stress and ROS accumulation. This likely resulted in decreased expression of detoxification-related and xenobiotic transport-related genes as Al toxicity would have less effect on *BnALMT7-A4*-overexpressing plants.

## Conclusion

We have identified a putative aluminium-activated malate transporter, *Bn*ALMT7-A4, that is induced by Al treatment in roots. We engineered *Arabidopsis* and *Brassica* overexpressing *Bn*ALMT7-A4 to generate Al-tolerant plants with improved root growth and reduced Al accumulation in root tips. Transcriptomic analysis of the Al-tolerant *Brassica* roots demonstrated modification of stress- and toxicity-specific gene expression. Thus, we have discovered a new way of making rapeseed, an important crop, more tolerant to Al stress.

## Data Availability

The data presented in the study are deposited in the China National Center For Bioinformation (https://ngdc.cncb.ac.cn), accession number CRA035735.
