# Supplementary material for: BnALMT7-A4 encodes an aluminium-activated malate transporter that enhances aluminium tolerance in both Brassica napus L. and Arabidopsis thaliana
Source: Front Plant Sci. 2026 Jan 28;16:1710318. doi: 10.3389/fpls.2025.1710318 (PMC12890689; doi:10.3389/fpls.2025.1710318)
Supplement: Supplementary file 1 [file DataSheet1.pdf]

Supplementary data

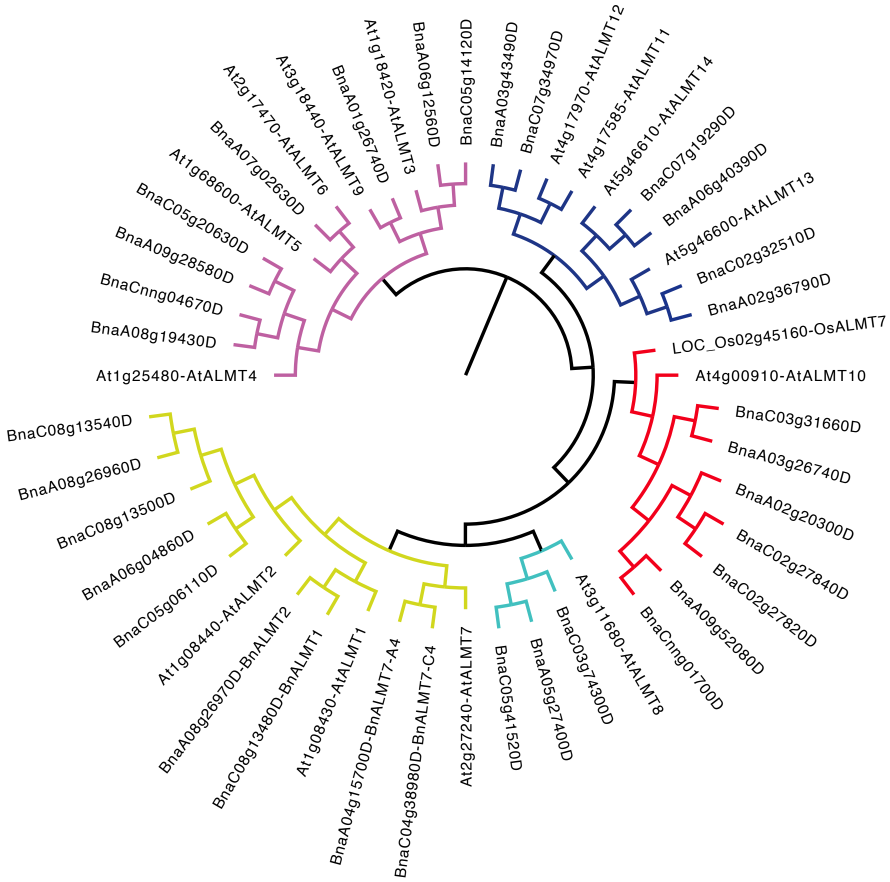

Figure S1. Phylogeny tree of Aluminum-activated malate transporter (*ALMT*) gene family in *Brassica napus* and *Arabidopsis thaliana*.

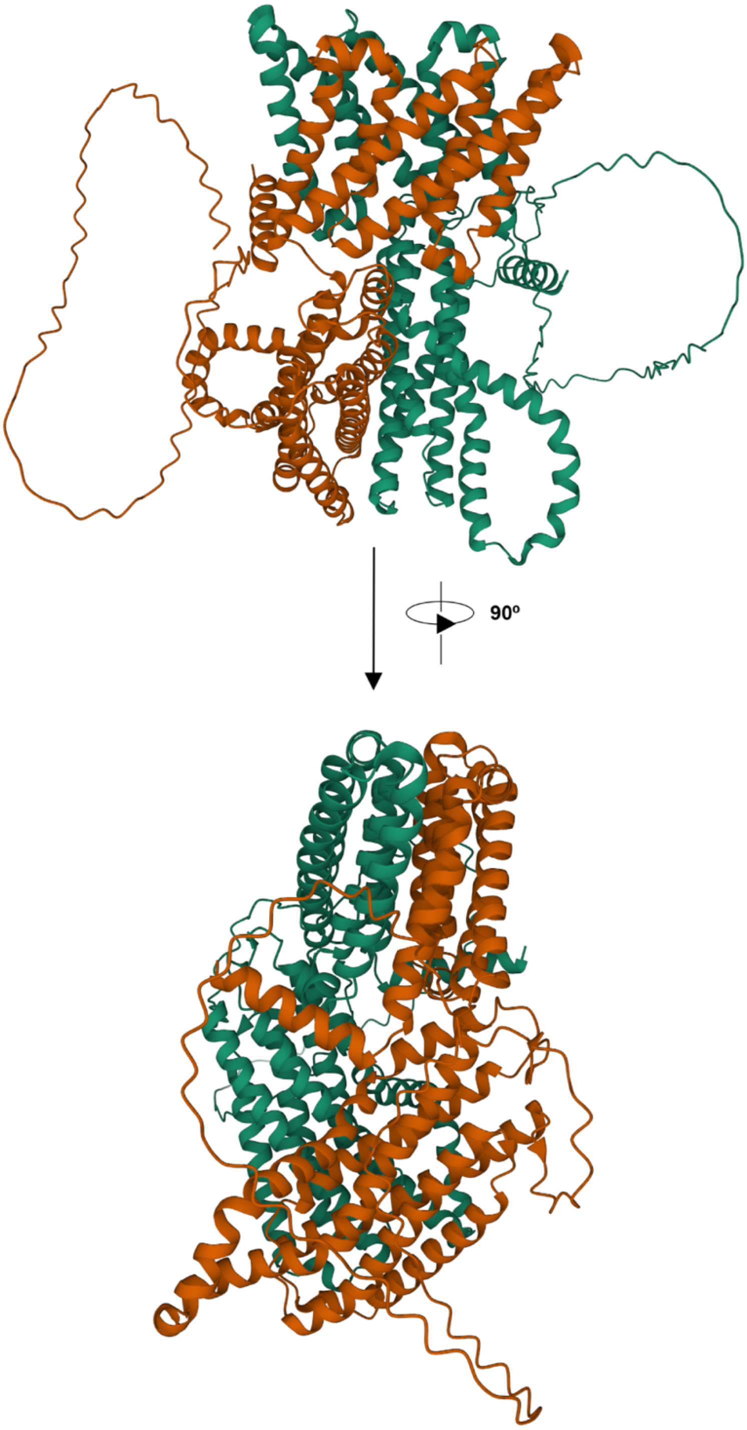

Figure S2. Prediction of BnALMT7-A4 homodimer structure by AlphaFold3

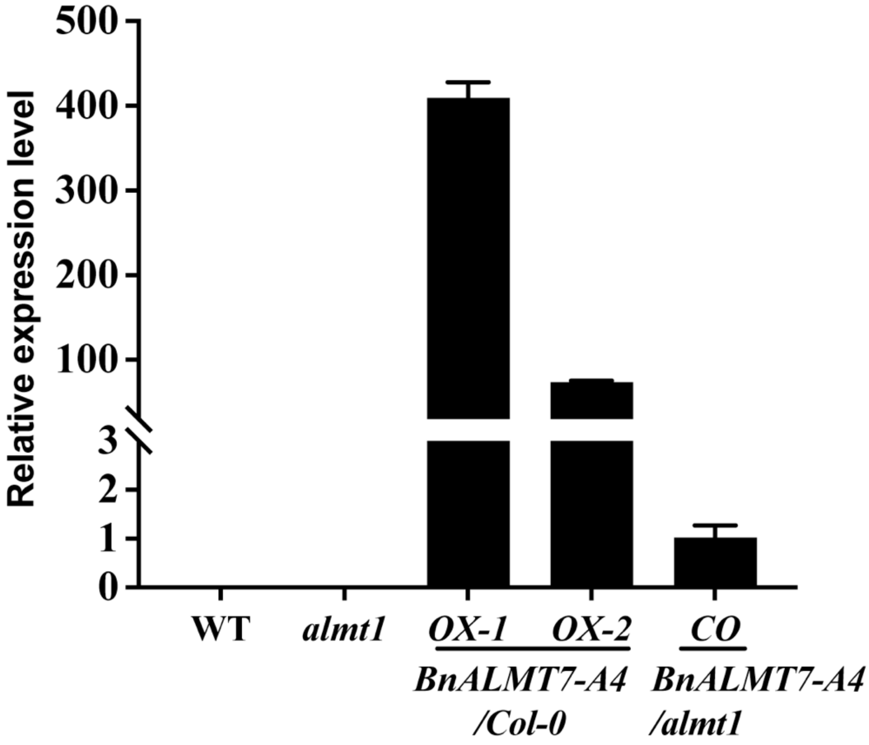

Figure S3. The relative expression of BnALMT7-A4 in transgenic Arabidopsis lines

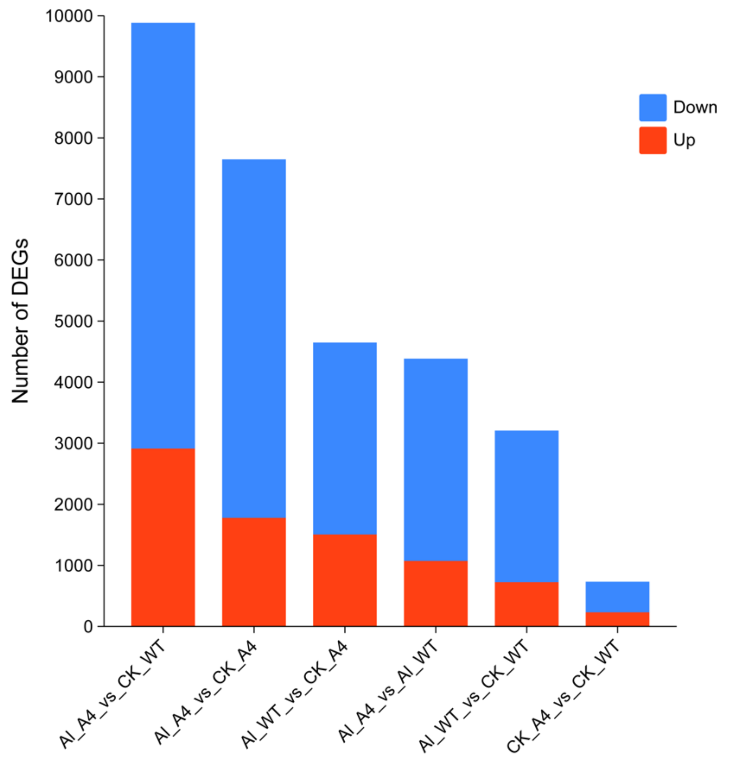

Figure S4. Numbers of DEGs in comparisons among CK_WT, CK_A4, Al_WT and Al_A4.

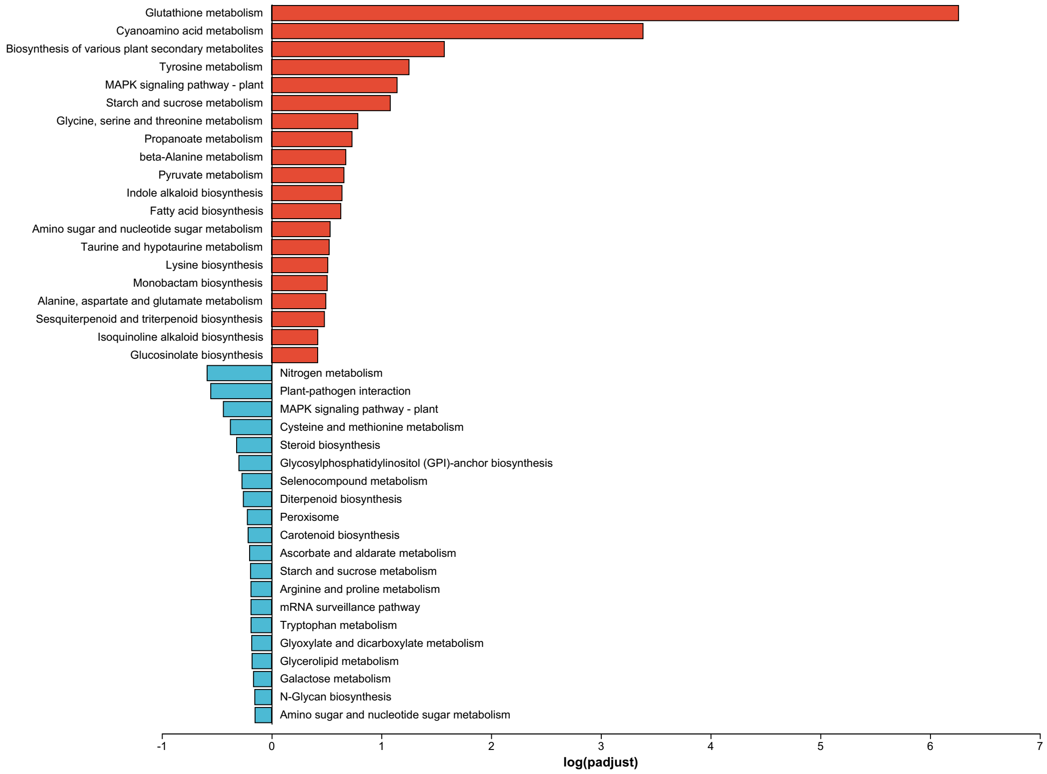

Figure S5. KEGG enrichment analysis of group-175 and group-219 from Venn Diagram analysis. Red bars indicate enriched KEGG pathways in group-175 and blue bars represent enriched KEGG pathways in group-219

Table S1 Primers for qRT-PCR.

Primer name	Primer Sequence	Primer name	Primer Sequence
BnALMT7-A4-qPCR-F	CCGATCTTCATGTCCGAAACGC	*BnALMT7-A4*-qPCR-R	GAGCAAAGAGATTGCTGTCAG
BnALMT7-C4-qPCR-F	TGCGGGGGGACTTGGTATCG	*BnALMT7-C4*-qPCR-R	CGCACAAATGTCGACAAGGCC
BnACTIN-F	CCTCTGCAGCCTCCTCAAGT	*BnACTIN*-R	CATATCTCCCCTGTCTTGAAATGC

Table S2 Primers for vector construction.

Primer name	Primer Sequence	Vector name
BnALMT7-A4-F	CCAAGCTTATGGAAAAAGTGAGAG	Pcx-DG
BnALMT7-A4-R	GCTCTAGATTATCTACATTCTACAA	Pcx-DG
BnALMT7-A4-GFP-F	AAAGGTACCATGGAAAAAGTGAGAGACCTC	pNM-GFP
BnALMT7-A4-GFP-R	AAAGTCGACTCTACATTCTACAATTACACGAA	pNM-GFP
p*BnALMT7-A4*-GUS-F	TACGAATTCGAGCTCGGTACCTACGATACGATTAATATTGTTATC	pNC-Cam1305.1
p*BnALMT7-A4*-GUS-R	TTACCCTCAGATCTACCATGGTTCTATCGATCTCTGTCTCAA	pNC-Cam1305.1
BnALMT7-A4-NLuc/CLuc -F	AGTGGTCTCTGTCCAGTCCTCAGTGCATTCTCCTCTGCCA	pNC-cam1300-NLuc/CLuc
BnALMT7-A4-NLuc/CLuc-R	GGTCTCAGCAGACCACAAGTTCTACATTCTACAATTACACGAA	pNC-cam1300-NLuc/CLuc
